# Clinical and dosimetric feasibility of sparing submandibular gland in patients with oral cavity squamous cell carcinoma

**DOI:** 10.1080/07853890.2024.2445186

**Published:** 2024-12-21

**Authors:** Yi-Peng He, Ping Zhou, Li-Mei Guan, San-Gang Wu

**Affiliations:** a Department of Radiation Oncology, Xiamen Cancer Center, Xiamen Key Laboratory of Radiation Oncology, The First Affiliated Hospital of Xiamen University, School of Medicine, Xiamen University, Xiamen, People’s Republic of China; b Department of Radiation Oncology, Fudan University Shanghai Cancer Center Xiamen Hospital, Xiamen, People’s Republic of China; c Department of Otolaryngology-Head and Neck Surgery, Xiamen Key Laboratory of Otolaryngology-Head and Neck Surgery, The First Affiliated Hospital of Xiamen University, School of Medicine, Xiamen University, Xiamen, People’s Republic of China

**Keywords:** Oral cavity cancer, submandibular gland, radiotherapy, distribution, treatment planning

## Abstract

**Background:**

To investigate the incidence of submandibular gland (SMG) involvement and explore the feasibility of sparing SMG in the oral cavity squamous cell carcinoma (OSCC).

**Methods:**

This study retrospectively analyzed patients between January 2018 to October 2022. Ten patients with tongue squamous cell carcinoma receiving postoperative radiotherapy were replanned to investigate the feasibility of sparing SMG. The dose constraint for the SMG was a mean dose (Dmean) <39 grey (Gy).

**Results:**

A total of 238 patients were identified and 105 had metastatic neck lymph nodes. Level II was the most common site of metastasis (*n* = 94, 89.5%), followed by level IB (*n* = 37, 35.2%), level III (*n* = 26, 24.8%), level IA (*n* = 3, 2.6%), and level IV (*n* = 2, 1.9%). A total of 50 metastatic lymph nodes were located at the level IB, of which 18 (36.0%), 29 (58.0%), and 3 (6%) were located in the lateral, anterior, and inferior aspect of the SMG. No metastatic lymph nodes were found within or on the medial aspect of the SMG. The Dmean of the SMG was <39 Gy in all patients with a Dmean of 38.8 Gy. The median dose of PTV54 D95% was 53.8 Gy, which met the prespecified allowable coverage goal.

**Conclusions:**

Our study suggests that SMG involvement is rare in OSCC. With strict imaging and clinical evaluation, sparing SMG during radiotherapy is feasible.

## KEY MESSAGES

1. We explored the feasibility of sparing SMG during radiotherapy in OSCC.
2. SMG involvement is rare in OSCC
3. Sparing SMG during radiotherapy is feasible.

## Introduction

Oral cavity cancer is a common type of tumor in the head and neck region. It is estimated that there are approximately 370,000 new cases of lip and oral cancer each year, accounting for about 2% of all malignant tumors [1]. Approximately 90% of oral cavity cancer patients are diagnosed with squamous cell carcinoma (SCC) [2]. Surgery combined with radiotherapy and/or chemotherapy is the main treatment modality for oral cavity SCC (OSCC). The rate of neck lymph node metastasis in OSCC is approximately 29-36% [3,4], which is closely related to the primary tumor site and tumor stage. Even for early-stage OSCC, the probability of occult neck lymph node metastasis is still around 20% [5]. Levels I-III are the most common sites of lymph node metastasis in OSCC [6]. The clinical management of neck lymph nodes is mainly determined based on patients’ pre-treatment neck lymph node status [7].

The submandibular gland (SMG) is the second largest major salivary gland. About 65% of unstimulated (resting) saliva comes from the SMG. In the stimulated state, the saliva production from the parotid and the SMG is approximately the same. According to the 2013 edition of the international consensus guidelines of neck node levels for head and neck tumors, the SMG is one of the contents in level IB [8]. In current clinical practice, neck lymph node dissection for OSCC usually involves the removal of the SMG located within level IB [9]. For OSCC patients undergoing definitive or postoperative radiotherapy, the level IB also needs to be irradiated, and the SMG is often located within the irradiation field [10]. However, there has been an ongoing debate about whether the SMG should be included during surgery or radiotherapy. A systematic review study including 2750 patients of OSCC found an incidence rate of SMG involvement of 2.05% [11]. Additionally, studies based on embryonic development suggest that there are no lymphatic channels or lymph nodes within the SMG [12,13]. Therefore, further research is warranted regarding the management of SMG in OSCC patients without SMG involvement. In this study, we aimed to investigate the incidence of SMG involvement and explore the feasibility of sparing SMG when irradiating level IB in the treatment of OSCC.

## Materials and methods

### Patients

This study retrospectively collected data from patients with OSCC who received surgical treatment at the First Affiliated Hospital of Xiamen University from January 2018 to October 2022. Patients who met the following criteria were included in the study: 1) tumor originating in the oral cavity, including the lip, maxilla, buccal mucosa, floor of mouth, retromolar trigone, tongue, or gingiva; 2) pathologically confirmed stage T1-4N0-3M0 OSCC; 3) complete clinical and pathological data; 4) underwent curative surgical resection of the primary tumor with ipsilateral or bilateral neck lymph node dissection including level IB and SMG. The following patients were excluded: 1) those who had previously undergone head and neck surgery, preoperative chemotherapy, immunotherapy, or preoperative radiotherapy; 2) those with a history of other head and neck tumors that may involve the SMG. This study was approved by the Institutional Review Boards of the First Affiliated Hospital of Xiamen University, and written informed consent for data use was obtained from all patients (approval number: 2023050).

### Treatment principle

In our institution, the treatment approach for individuals with OSCC involves surgical removal of the primary tumor with ipsilateral or bilateral neck lymph node dissection. The primary tumor was removed with at least a 1 cm margin and reconstruction with a flap was performed if necessary. A therapeutic neck dissection included a minimum of levels IA, IB, II, III, and IV. Due to the rare occurrence of nodal metastasis in level V, level V was not routinely dissected in OSCC. However, level V dissection should be included in patients with bulky or multilevel nodal disease. Bilateral neck dissection was performed in patients with tumors abuts or crosses the midline. The need for adjuvant therapy was determined according to the individual risk factors of each patient.

### Variables

We collected the following demographic, clinical, and pathological characteristics for analysis: age, gender, primary tumor location, smoking index (0, ≤20, or >20), history of alcohol consumption, tumor grade, pathological tumor (T) staging, pathological nodal (N) staging, American Joint Committee on Cancer (AJCC) staging, surgical margin status, neck lymph node dissection, involvement of the SMG. The staging was performed according to the 8th edition of the AJCC staging for OSCC [14]. The neck lymph node levels were defined according to the 2013 edition of the international consensus guidelines of neck node levels for head and neck tumors [8].

### Radiation therapy planning

We conducted an additional dosimetric study on 10 patients with tongue SCC who underwent curative surgical resection of the primary tumor and ipsilateral neck lymph node dissection to explore the feasibility of sparing SMG when irradiating level IB in the treatment of patients. As the ipsilateral SMG was already removed during neck lymph node dissection, we focused on studying the sparing of the contralateral SMG.

Target volume delineation: The target volume delineation was performed according to the consensus and guidelines for head and neck tumor delineation [8,15]. We mainly delineated the high-risk clinical target volume (CTV60) and low-risk CTV (CTV54). CTV60 included the entire tongue, floor of mouth, and ipsilateral neck lymph nodes in levels I-IV. CTV54 mainly included the contralateral neck lymph nodes in levels I-IV, while avoiding the delineation of contralateral SMG. A margin of 0.3 cm was added around the CTV to create the planning target volume (PTV). The target volume delineation for all 10 patients was performed by an experienced head and neck radiation oncologist (SGW).

Prescription dose and normal organ dose limits: The prescription dose for high-risk PTV (PTV60) was 60 grey (Gy) delivered in 2 Gy per fraction, while the prescription dose for low-risk PTV (PTV54) was 54 Gy delivered in 1.8 Gy per fraction. The dose limits for organs at risk and their priorities were referenced from previous international guidelines [16]. Based on the recommendations from international guidelines and the study by Murdoch-Kinch et al. we restricted the mean dose (Dmean) to the SGM of <39 Gy [16,17]. We cropped out the SMG from PTV54 and used the tailored volume (PTV54-SMG) for treatment planning optimization. The minimum dose (Dmin), maximum dose (Dmax), Dmean, and dose received by 95% of the volume (D95%) were assessed. Dmin refers to the minimum dose received by any part of the target volume, Dmax refers to the maximum dose received by any part of the target volume, and Dmean refers to the average dose received by the entire target volume.

Treatment planning and evaluation: All radiotherapy plans were regenerated using Eclipse treatment planning system version 15.6 for a Truebeam linear accelerator (Varian Medical Systems, Inc., Palo Alto, CA) using flattening filter free volumetric modulated arc therapy (VMAT-FFF) with three to four coplanar full arcs and 6 MV photons. Photon optimizer algorithm and anisotropic analytical algorithm (both version 15.6, Varian Medical Systems, Inc., Palo Alto, CA) were employed for planning optimization and final dose calculation, respectively. The target doses for PTV54 were set to achieve a D95% greater than 54 Gy, with an allowable variation of greater than 48.6 Gy, as specified by Radiation Therapy Oncology Group (RTOG) 1008 [18]. Dosimetric information, including volumes, Dmin, Dmax, and Dmean of the SMG, PTV54, PTV associated with level IB only (PTV_IB), and CTV associated with level IB only (CTV_IB), as well as the doses received by 95% of the target volumes (PTV54 D95%, PTV_IB D95%, CTV_IB D95%), were collected from the dose-volume histograms (DVHs) in the treatment planning system for each patient.

### Follow-up

In this study, survival data were retrospectively collected by reviewing medical records. All patients underwent regular follow-up assessments at three-month intervals for a minimum of three years. These assessments included monitoring physical examination and CT or MRI of the head and neck. Immediate imaging examinations, such as PET/CT, were conducted for patients exhibiting signs of disease progression. Disease-free survival (DFS) was the primary endpoint in this study, which defined as the time from diagnosis to the first occurrence of locoregional recurrence (LRR), distant metastasis (DM), or death from any cause.

### Statistical analysis

The demographic and pathological characteristics were analyzed using descriptive analysis. The survival curves were plotted using the Kaplan-Meier method. Statistical analyses were performed using SPSS 26.0 (IBM Corporation, Armonk, NY, USA) software.

## Results

### Patient characteristics

A total of 238 OSCC patients were included in this study. The patient baseline characteristics are shown in Table 1. Most of these patients were male (*n* = 154, 64.7%). The primary tumors were mostly located in the tongue (*n* = 155, 65.1%), buccal mucosa (*n* = 45, 18.9%), and floor of the mouth (*n* = 19, 8.0%). According to the 8th edition of the AJCC staging criteria, 52 (21.8%), 57 (23.9%), 44 (18.5%), 64 (26.9%), and 21 (8.8%) were diagnosed as stage I, II, III, IVA, and IVB diseases, respectively. A total of 195 patients (81.9%) underwent unilateral neck lymph node dissection and 43 patients (18.1%) had bilateral neck lymph node dissection.

**Table 1.** Patient baseline characteristics (*n* = 238).

Variables	*N* (%)
Gender	
Male	154 (64.7)
Female	84 (35.3)
Age (years)	
<50	51 (21.4)
≥50	187 (78.6)
Tumor location	
Lip	1 (0.4)
Upper jaw	3 (1.3)
Buccal mucosa	45 (18.9)
Mouth floor	19 (8.0)
Retromolar trigone	1 (0.4)
Tongue	155 (65.1)
Gingiva	14 (5.9)
Smoking pack-year index	
0	136 (57.1)
≤20	18 (7.6)
>20	87 (35.3)
Alcohol use	
Never	131 (55.0)
Normal	51 (21.4)
Abuse	56 (23.5)
Tumor grade	
Well differentiation	34 (14.3)
Moderate differentiation	178 (74.8)
Poor differentiation	26 (10.9)
Tumor stage	
T1	72 (30.3)
T2	110 (46.2)
T3	35 (14.7)
T4	21 (8.8)
Nodal stage	
N0	133 (55.9)
N1	30 (12.6)
N2	54 (22.7)
N3	21 (8.8)
AJCC stage	
I	52 (21.8)
II	57 (23.9)
III	44 (18.5)
IVA	64 (26.9)
IVB	21 (8.8)
Margin status	
Negative	224 (94.1)
Positive	14 (5.9)
Neck lymph node dissection	
Ipsilateral	195 (81.9)
Bilateral	43 (18.1)
Submandibular gland involved	
No	235 (98.7)
Yes	3 (1.3)

### Distribution of metastatic neck lymph nodes

**Figure 1.**
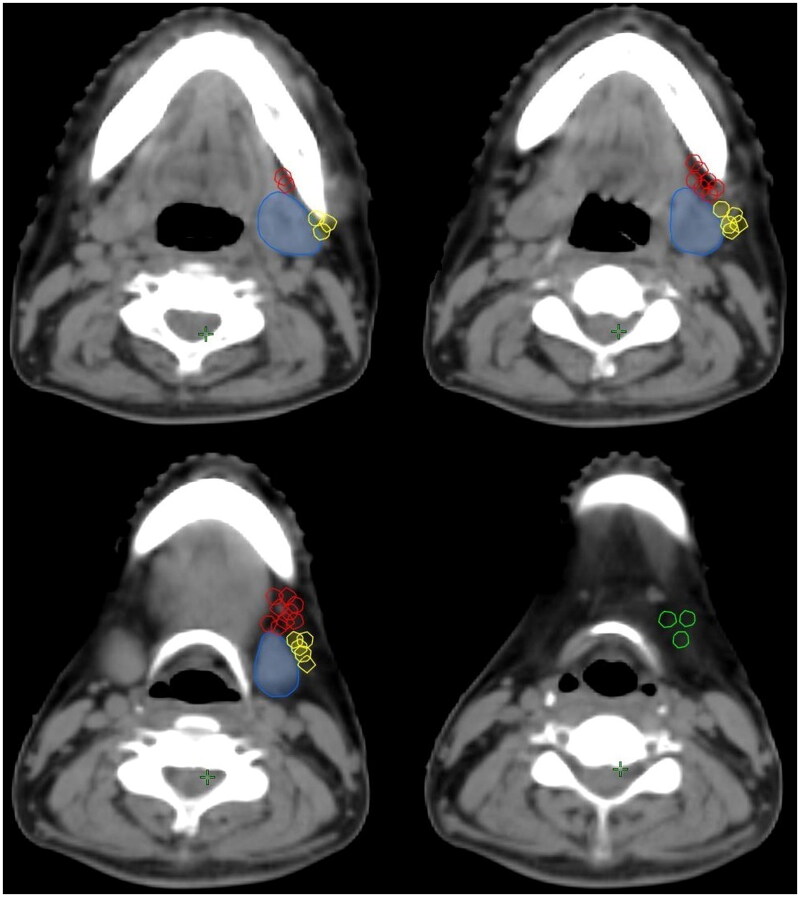
The distribution of level IB lymph node metastasis in oral squamous cell carcinoma (blue line, submandibular gland; red line, lymph node metastases anterior to the submandibular gland; yellow line, lymph node metastases on the lateral side of the submandibular gland; green line, lymph node metastases below the submandibular gland).

A total of 105 patients were pathologically confirmed to have metastatic neck lymph nodes, with level II being the most common site of metastasis (*n* = 94, 89.5%), followed by level IB (*n* = 37, 35.2%), level III (*n* = 26, 24.8%), level IA (*n* = 3, 2.6%), and level IV (*n* = 2, 1.9%). Among the 37 patients with level IB lymph node metastasis, a total of 50 metastatic lymph nodes were identified, of which 18 were located in the lateral aspect of the SMG (36.0%), 29 were located in the anterior aspect of the SMG (58.0%), and 3 were located in the inferior aspect of the SMG (6%). No metastatic lymph nodes were found within the SMG or on the medial aspect of the SMG. Figure 1 shows the patterns of level IB metastatic lymph nodes in the 37 patients with level IB lymph node metastasis.

**Figure 2.**
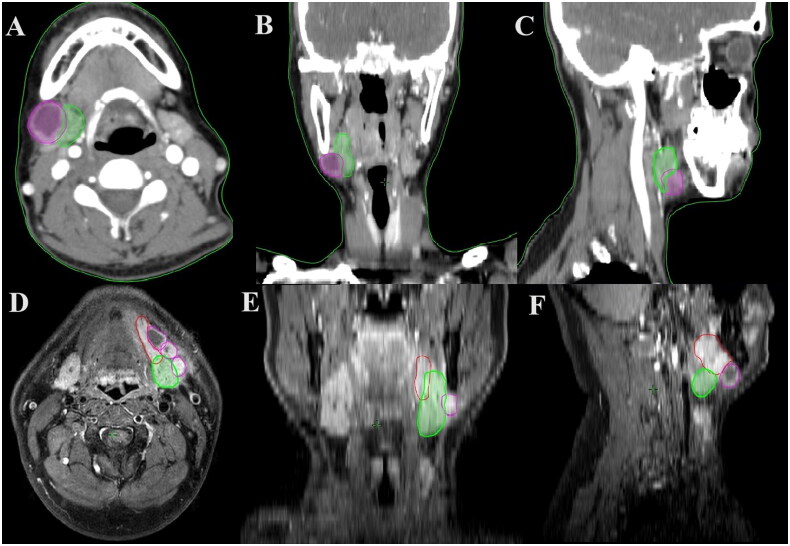
Two oral squamous cell carcinoma patients with preoperative imaging involving the submandibular gland (A-C: preoperative enhanced CT and postoperative pathology showed extra-capsular invasion of the submandibular gland by metastatic lymph nodes in the level IB in a patient with stage T1N3bM0 IVB squamous cell carcinoma of the right buccal mucosa [A: axial view, B: coronal view, C: sagittal view]. D-F: preoperative enhanced MRI and postoperative pathology showed invasion of the submandibular gland by metastatic lymph nodes in the level IB in a patient with stage T2N3bM0 IVB left tongue squamous cell carcinoma. The preoperative enhanced MRI showed indistinct boundaries between the primary tumor and the submandibular gland and sublingual gland in this patient [D: axial view, E: coronal view, F: sagittal view]). (red line, primary tumor; green line, submandibular gland; purple line, lymph node metastasis in level IB).

Among these patients, three patients (1.3%) were pathologically confirmed to have involvement with the SMG. The first patient had stage T4bN3bM0 IVB tongue SCC, preoperative enhanced computed tomography (CT), and postoperative pathology confirmed that the primary tumor directly infiltrated the SMG. The second patient had stage T1N3bM0 IVB right buccal mucosa SCC, preoperative enhanced CT, and postoperative pathology indicated that the metastatic lymph nodes with extranodal extension in level IB invaded the SMG (Figure 2A–C). The third patient had stage T2N3bM0 IVB tongue SCC. Preoperative enhanced magnetic resonance imaging (MRI) and postoperative pathology showed that the metastatic lymph node with extranodal extension in level IB invaded the SMG. Contrast-enhanced MRI showed indistinct boundaries between the primary tumor and the SMG and sublingual gland (Figure 2D–F).

### Dosimetric analysis

**Figure 3.**
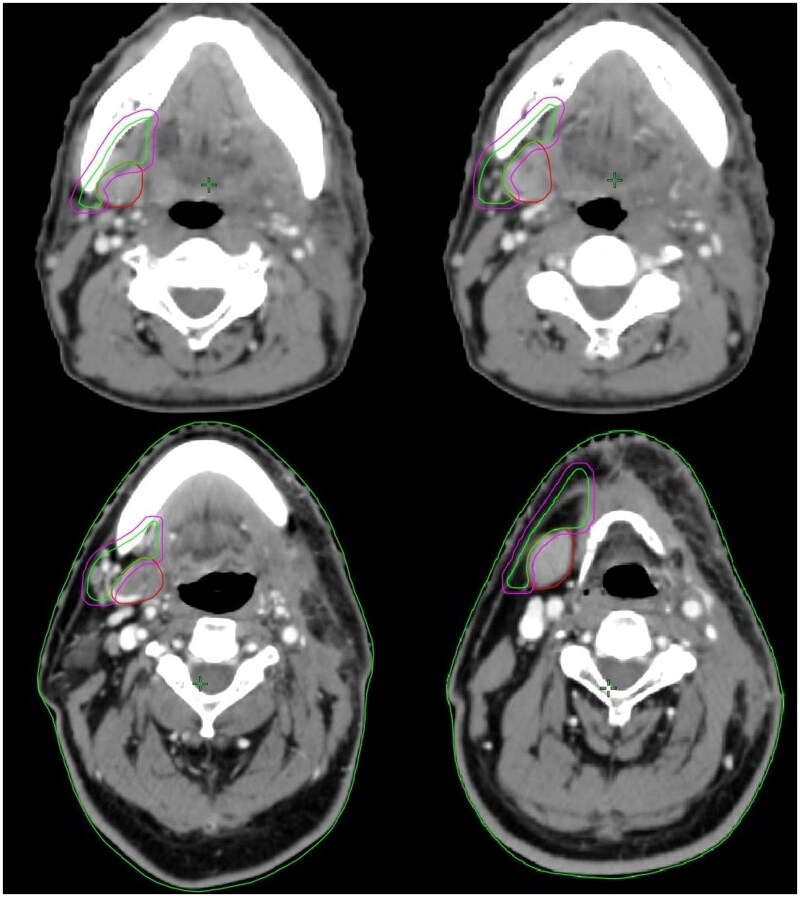
Representative contralateral level IB delineation in a patient with oral squamous cell carcinoma with ipsilateral neck lymph node dissection and bilateral neck lymph node irradiation (red line, submandibular gland; green line, CTV_IB; purple line, PTV_IB).

**Figure 4.**
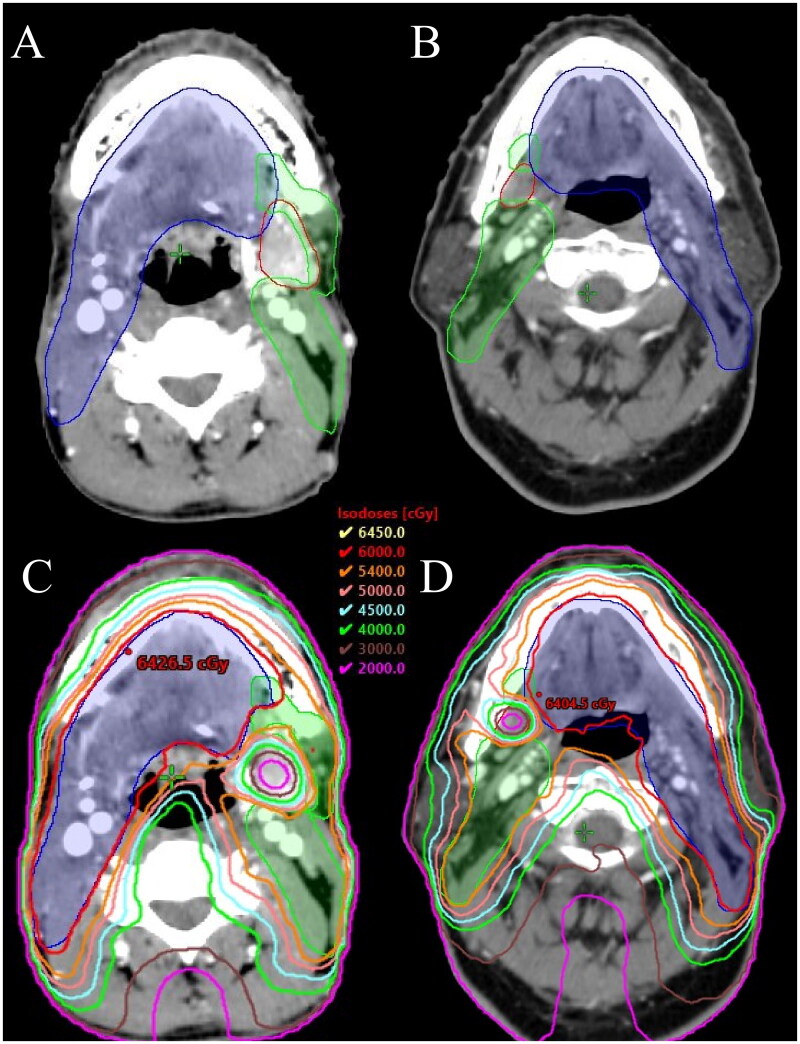
Representative target volumes (a and B: green line, PTV54; blue line, PTV 60; red line, submandibular gland) and dose distribution (C and D) in two patients.

We conducted the dosimetric study on 10 tongue SCC patients who underwent surgical resection of the primary tumor and ipsilateral neck lymph node dissection to explore the feasibility of sparing contralateral SMG when irradiating level IB in the patients. Figure 3 lists the contouring of the target volume of sparing contralateral SMG in two patients with tongue SCC. The SMG was excluded from the CTV_IB, but a 3 mm expansion would include a portion of the SMG in the PTV_IB. Figure 4A,B show the contouring of the target volume in two patients for treatment planning. Table 2 lists the Dmin, Dmax, and Dmean of the SMG for ten patients. Table 3 presents the PTV54 volume, Dmin, Dmax, Dmean, and D95% of the PTV54 for ten patients. The average volume of the SMG was 8.5 cubic centimeters (cc) (range, 3.9-14.2 cc). The Dmean of the SMG was <39 Gy in all patients with a Dmean of 38.8 Gy (range, 38.7-38.9 Gy). The Dmean of PTV54 was 56.7 Gy (range, 56.0-57.1 Gy). The median dose of PTV54 D95% was 53.8 Gy (range, 52.8-54.5 Gy), which met the prespecified allowable coverage goal.

**Table 2.** Summary of dose to the submandibular gland.

Patient	volume (cc)	Dmin (Gy)	Dmean (Gy)	Dmax (Gy)
1	10.5	14.7	38.7	57.3
2	4.0	20.7	38.8	53.9
3	3.9	20.2	38.9	55.0
4	9.5	12.7	38.8	59.1
5	10.0	12.4	38.9	61.0
6	7.6	15.4	38.8	58.6
7	9.8	14.6	38.9	57.6
8	6.4	14.9	38.9	58.2
9	8.8	15.1	38.9	56.6
10	14.2	13.7	38.7	59.4

**Table 3.** Summary of dose to the PTV54.

Patient	Volume (cc)	Dmin (Gy)	Dmean (Gy)	Dmax (Gy)	D95% (Gy)
1	202.1	35.2	56.5	64.1	53.9
2	186.8	31.0	56.0	63.1	53.5
3	156.3	28.8	56.7	62.7	54.2
4	145.9	35.8	57.1	63.5	54.5
5	194.9	31.1	56.8	63.8	53.8
6	148.8	32.6	56.9	64.0	53.7
7	232.2	31.1	56.8	63.1	53.8
8	137.2	31.7	56.7	63.7	53.7
9	198.7	30.3	56.5	63.3	52.8
10	264.9	35.8	56.8	63.3	54.3

The Dmean of PTV_IB was 56.9 Gy (range, 56.5-57.4 Gy), and the median dose of PTV_IB D95% was 50.6 Gy (range, 48.6-52.5 Gy) (Table 4). Figure 4C,D show the isodose curves for two patients. The Dmean of CTV_IB was 57.7 Gy (range, 57.1-58.3 Gy), and the median dose of CTV_IB D95% was 54.0 Gy (range, 53.2-55.3 Gy) (Table 5). The sparing of the SMG resulted in an underdosing of approximately 2.8-9.9% of the part of the PTV that corresponds to level IB. This slight underdosing occurred due to a small portion of the PTV lying within the SMG (Table 6).

**Table 4.** Summary of dose to the PTV_IB.

Patient	Volume (cc)	Dmin (Gy)	Dmean (Gy)	Dmax (Gy)	D95% (Gy)
1	32.4	35.2	56.5	64.1	51.4
2	32.3	33.9	56.8	63.1	49.2
3	22.1	28.8	56.7	62.7	48.6
4	18.3	36.0	57.2	63.5	51.4
5	28.6	31.1	57.1	63.8	50.7
6	24.7	32.8	56.9	64.0	50.2
7	40.4	36.6	57.3	63.1	51.0
8	32.7	36.1	56.9	63.7	50.6
9	41.2	30.3	56.7	63.3	50.4
10	30.3	40.5	57.4	63.3	52.5

**Table 5.** Summary of dose to the CTV_IB.

Patient	Volume (cc)	Dmin (Gy)	Dmean (Gy)	Dmax (Gy)	D95% (Gy)
1	12.6	51.2	57.1	62.1	53.5
2	12.7	47.7	57.5	63.0	53.2
3	8.4	43.9	57.3	62.7	53.3
4	5.5	48.7	58.2	62.9	54.8
5	11.3	49.1	58.1	63.8	54.2
6	7.6	48.9	57.8	62.5	54.1
7	16.1	51.5	57.9	63.1	54.2
8	13.3	50.1	57.5	63.5	53.9
9	16.7	48.0	57.4	62.4	53.5
10	10.2	52.4	58.3	63.2	55.3

**Table 6.** PTV_IB volumes in the SMG.

Patient	Volume of PTV_IB (cc)	Volume of PTV_IB within SMG (cc)	Ratio (%)
1	32.4	2.4	7.5
2	32.3	1.2	3.7
3	22.1	1.1	5.1
4	18.3	2.2	11.9
5	28.6	2.2	7.7
6	24.7	1.7	7.0
7	40.4	2.9	7.2
8	32.7	1.9	5.8
9	41.2	2.6	6.2
10	32.4	2.6	9.3

### Patterns of disease recurrence

The median follow-up time was 46.8 months (range, 4-84 months). Among all patients, 72 experienced a DFS event (30.3%), which included 42 patients with LRR alone (58.3%), 14 patients with both LRR and DM (19.4%), 10 patients with DM alone (13.9%), and 6 deaths due to other causes (8.3%). The 1-year, 3-year, and 5-year DFS rates for all patients were 81.9%, 72.1%, and 68.9%, respectively.

Of the 56 patients who experienced LRR with or without DM, 34 had recurrence in the neck (60.7%), 14 had local recurrence (25.0%), and 8 had both local and regional recurrence (14.3%).

Among those with recurrence in the cervical lymph nodes (*n* = 42), 15 had recurrence in the ipsilateral level IB lymph nodes (35.7%), 4 had bilateral level IB lymph node recurrence (9.5%), and 1 had contralateral level IB lymph node recurrence (2.4%). As the ipsilateral SMG was removed during surgery, recurrence at this site could not be evaluated. Among the 5 patients with contralateral level IB lymph node recurrence, recurrence sites were located anterior or lateral to the SMG, with no intraglandular recurrence observed.

## Discussion

In this study, we aimed to explore the clinical and dosimetric feasibility of sparing SMG during radiotherapy in patients with OSCC. We found that the incidence of SMG involvement in OSCC was only 1.3%, mainly due to extracapsular spread of adjacent metastatic lymph nodes or direct invasion of the primary tumor. Furthermore, we found that it is feasible to spare the SMG in postoperative radiotherapy for tongue SCC, with the Dmean of the SMG kept below 39 Gy while ensuring acceptable target volume coverage.

Most patients with OSCC who undergo surgical treatment also require neck lymph node dissection. Despite multiple modifications to neck lymph node dissection based on a better understanding of the patterns of lymph node metastasis, it is still generally recommended to remove the SMG as part of the level IB dissection [9,19,20]. For patients with tumors crossing the midline or bilateral neck lymph node metastasis, bilateral SMG will be removed. However, several studies have demonstrated that the incidence of SMG in OSCC ranges from 0-4.5% [11,21,22]. In our study, the incidence of SMG involvement was only 1.3%. Our study also did not find any lymph node recurrence located within the SMG in patients with cervical lymph node recurrence. Therefore, it is clinically feasible to preserve the uninvolved SMG during the surgery or radiotherapy of OSCC. There are three potential modes of SMG involvement in OSCC: hematogenous spread, lymphatic spread, and anatomical direct invasion [23]. The presence of lymph nodes within the SMG is still controversial in OSCC. Potential hematogenous spread to the SMG has been reported in other cancers [24], but it is difficult to distinguish whether SMG in OSCC is due to tumor metastasis or anatomical proximity [23,25,26]. In our study, all three patients of SMG were caused by tumor invasion or extracapsular spread of lymph nodes within the level IB. A literature review including 2750 patients found SMG in 59 patients (2.05%), with 44 (74.5%) being direct invasion by the primary tumor, 13 (22.0%) being the invasion of lymph nodes around the SMG, and 2 (3.4%) being lymph node metastasis within the SMG [11].

Several studies based on histological embryology suggest that the development of the SMG occurs earlier than the development of the lymphatic system, and the neck fascia separates the parenchyma of the SMG from the lymph nodes [12,13]. Therefore, it is generally believed that there are no lymph nodes within the SMG. A large cohort study conducted on nasopharyngeal carcinoma found no evidence of metastatic lymph nodes within or on the medial aspect of the SMG [27]. In our study, all patients underwent neck lymph node dissection and concurrent removal of the SMG, but no metastatic lymph nodes were found within or on the medial aspect of the SMG. Our study, along with previous studies, suggests that direct invasion of lymph nodes in the vicinity of the SMG and periglandular metastatic lymph nodes is the main factor leading to SMG involvement [11,28–30]. We also found that the common sites of metastasis in OSCC were level II, level IB, and level III. Since SMG involvement is easily detectable in imaging studies, it may be feasible to preserve the SMG or avoid irradiation of the SMG during surgery or radiotherapy if there is no evidence of SMG involvement.

The SMG has the main function of saliva secretion, with approximately 70% of unstimulated saliva being produced by the SMG, especially during the night [31]. Jaguar et al. assessed the impact of SMG removal on salivary gland function [32]. The results showed a significant reduction in postoperative unstimulated saliva flow rate, while stimulated saliva flow rate remained unchanged. Additionally, the removal of the SMG may also result in external contour defects in the neck, affecting aesthetics [33]. According to guidelines, the SMG is one of the contents in the level IB, which is inevitably irradiated in patients receiving postoperative radiotherapy. Therefore, in OSCC patients undergoing adjuvant radiotherapy, the unavoidable irradiation of the contralateral SMG may further increase the incidence and severity of xerostomia. Bo et al. performed a study on 31 patients of early-stage buccal mucosal SCC with preserved SMG during neck lymph node dissection, and the results showed that preserving the SMG did not affect the local control rate and disease-specific survival [21]. Chen et al. conducted a study involving 408 patients with early-stage OSCC and similarly found that preserving the SMG did not increase the local regional recurrence rate and mortality rate in patients [34].

The widespread adoption of IMRT (intensity-modulated radiation therapy) technology has significantly minimized the radiation dose to the salivary glands, thus reducing the risk of xerostomia [35–38]. However, current research primarily focuses on avoiding the parotid glands using IMRT, and there is limited research on avoiding irradiation of the SMG in OSCC patients [35]. A prospective study conducted by Wang et al. concluded that patients with head and neck tumors who avoided irradiation of the SMG during postoperative radiotherapy had better recovery of salivary function and lower severity of xerostomia compared to those who received irradiation to SMG [39]. Varra et al. attempted to avoid irradiation of the SMG in patients with oral or oropharyngeal cancer and did not observe an increase in recurrence rates in the level IB. However, the Dmean to the SMG in the sparing and non-sparing groups were 58.9 and 66.6 Gy, respectively [40]. The irradiation dose to the SMG in the sparing group was much higher than the currently recommended dose limits for SMG (Dmean <39 Gy) [16,17]. Murdoch-Kinch et al. found that when the Dmean to the SMG was limited to <39 Gy, salivary function gradually recovered after radiotherapy [17]. Due to the target volume around the contralateral SMG being a major target volume for definitive radiotherapy or postoperative radiotherapy in OSCC, especially tongue cancer, avoiding irradiation of the SMG poses certain challenges.

In the current series, SMG involvement was observed in patients with T4 tumors or extranodal invasion of level IB lymphadenopathy. None of the patients with T1/T2 and node-negative disease showed SMG involvement. The findings of our study were consistent with a previous study [41]. The low incidence of SMG involvement in this series also supports the notion that routine resection may be an overtreatment and preservation should be considered for certain patients. However, in patients with large tumors and extensive level IB lymphadenopathy, SMG preservation may be technically challenging and raise concerns about oncological safety. Moreover, preserving SMGs in advanced OSCC can be complicated by the potential for dysfunction resulting from adjuvant radiotherapy, which is standard treatment in such cases. However, Nevertheless, advancements in radiotherapy techniques may offer the possibility of sparing the SMG during treatment planning to reduce morbidity in the future [42]. Therefore, SMG preservation may be a feasible option for patients without potential invasion of the primary tumor in adjacent areas or extranodal involvement of level IB lymphadenopathy. Furthermore, advancements in radiotherapy have made SMG-sparing post-operative radiation feasible.

VMAT is an advanced form of IMRT that enables precise dose delivery outside the target area in patients with complex target geometries by continuously adjusting beam fluences in a rotating manner. The use of FFF beams can further enhance treatment plan quality. A study by Jackson et al. demonstrated the feasibility of VMAT in SMG sparing in twelve oral cancer patients requiring irradiation of the contralateral level IB. They optimized their plans using FFF beams and observed a 10% to 20% underdosing in the PTV_IB, with a median D95% of 43.3 Gy (range, 42.5-52.2 Gy) [43]. In our study, we set a threshold of Dmean <39 Gy with VMAT-FFF, and all patients met the dose constraints, although there may have been approximately 2.8-9.9% underdosing in the PTV-IB area. The median dose of PTV_IB D95% was 50.6 Gy, which was deemed clinically acceptable according to RTOG 1008 [18] and RTOG 1016 guidelines [44]. Given the better immobilization techniques used in radiotherapy of head and neck cancers and the smaller potential errors compared to thoracic and abdominal tumors, we believe the potential underdose in the PTV-IB area is feasible.

We needed to acknowledge several limitations of our study. First, this is a retrospective study with a relatively small sample size. Second, our study did not collect data on the occurrence of xerostomia in patients who underwent SMG removal during neck lymph node dissection. Third, the data regarding recurrence/metastasis to the SMG and the survival data were not analyzed in our study.

Thirdly, further prospective studies are needed to evaluate the feasibility and safety of sparing SMG during radiotherapy in patients with OSCC.

## Conclusions

Our study suggests that SMG involvement is rare in OSCC. With strict imaging and clinical evaluation, sparing SMG during radiotherapy is feasible. Further prospective studies are needed to confirm our findings and explore the impact of sparing SMG on the development of xerostomia in OSCC.

## Data Availability

The datasets used and/or analyzed during the current study are available from the corresponding author upon reasonable request.
